# Environmental Modulation of Early Tau Pathology in Alzheimer's Disease: A Pretangle‐Tau Rat Model

**DOI:** 10.1002/alz70855_096920

**Published:** 2025-12-23

**Authors:** Zia Hasan, Sarah E Torraville, Tamunotonye Omoluabi, Aida Maziar, Onyedikachi N Belolise, Lauren A MacGowan, Cassandra M Flynn, Touati Benoukraf, Qi Yuan

**Affiliations:** ^1^ Memorial University of Newfoundland, St. John's, NF, Canada

## Abstract

**Background:**

Longitudinal studies implicate environmental factors as critical modulators of Alzheimer's disease (AD) progression. The locus coeruleus (LC), an early site of hyperphosphorylated tau accumulation, provides a unique target for intervention prior to the emergence of neurofibrillary tangles and amyloid plaques.

**Method:**

Using a rat model expressing pseudophosphorylated human tau (htauE14) in LC neurons, we explored how early‐ and late‐life environmental enrichment or stress affect tau pathology and behavioral outcomes.

**Result:**

HtauE14 expression heightened anxiety and impaired olfactory discrimination and spatial learning compared to GFP controls. Early enrichment mitigated tau spread, reduced LC inflammation, and elevated hippocampal BDNF levels, while late‐life enrichment reduced anxiety and improved exploratory behavior without altering tau pathology. Conversely, late‐life stress amplified LC inflammation. Single‐nucleus transcriptomics identified widespread htauE14‐induced gene expression changes, exacerbated by early stress in female hippocampi, alongside protective effects of early enrichment across neuronal and glial populations.

**Conclusion:**

These findings underscore the timing‐dependent and sex‐specific roles of environmental factors in AD pathogenesis, highlighting the importance of targeted interventions.

